# A Novel Translocation Breakpoint within the BPTF Gene Is Associated with a Pre-Malignant Phenotype

**DOI:** 10.1371/journal.pone.0009657

**Published:** 2010-03-11

**Authors:** Yosef Buganim, Ido Goldstein, Doron Lipson, Michael Milyavsky, Sylvie Polak-Charcon, Corine Mardoukh, Hilla Solomon, Eyal Kalo, Shalom Madar, Ran Brosh, Marina Perelman, Roy Navon, Naomi Goldfinger, Iris Barshack, Zohar Yakhini, Varda Rotter

**Affiliations:** 1 Department of Molecular Cell Biology, Weizmann Institute of Science, Rehovot, Israel; 2 Department of Computer Sciences, Technion—Institute of Technology, Haifa, Israel; 3 Department of Pathology, The Chaim Sheba Medical Center, Tel-Hashomer, Ramat Gan, Israel; 4 Agilent Technologies, Tel-Aviv, Israel; UMDNJ, United States of America

## Abstract

Partial gain of chromosome arm 17q is an abundant aberrancy in various cancer types such as lung and prostate cancer with a prominent occurrence and prognostic significance in neuroblastoma – one of the most common embryonic tumors. The specific genetic element/s in 17q responsible for the cancer-promoting effect of these aberrancies is yet to be defined although many genes located in 17q have been proposed to play a role in malignancy. We report here the characterization of a naturally-occurring, non-reciprocal translocation der(X)t(X;17) in human lung embryonal-derived cells following continuous culturing. This aberrancy was strongly correlated with an increased proliferative capacity and with an acquired ability to form colonies *in vitro*. The breakpoint region was mapped by fluorescence *in situ* hybridization (FISH) to the 17q24.3 locus. Further characterization by a custom-made comparative genome hybridization array (CGH) localized the breakpoint within the Bromodomain PHD finger Transcription Factor gene (BPTF), a gene involved in transcriptional regulation and chromatin remodeling. Interestingly, this translocation led to elevation in the mRNA levels of the endogenous BPTF. Knock-down of BPTF restricted proliferation suggesting a role for BPTF in promoting cellular growth. Furthermore, the BPTF chromosomal region was found to be amplified in various human tumors, especially in neuroblastomas and lung cancers in which 55% and 27% of the samples showed gain of 17q24.3, respectively. Additionally, 42% percent of the cancer cell lines comprising the NCI-60 had an abnormal BPTF locus copy number. We suggest that deregulation of BPTF resulting from the translocation may confer the cells with the observed cancer-promoting phenotype and that our cellular model can serve to establish causality between 17q aberrations and carcinogenesis.

## Introduction

Structural alterations in chromosomes (commonly termed aneuploidy or chromosomal aberrations) have been repeatedly reported to associate with malignancy. Although not in all cases a causal relationship has been proven, it is clear that abnormal rearrangements of chromosomes during mitosis can lead to carcinogenic events by deregulating proliferation-controlling pathways. Genetic alterations could pave the path leading to malignancy by several ways: in the most straight forward manner loss of a tumor suppressor gene (by deletion) or gain of an oncogene (by amplification) could unleash a proliferative pathway. A prominent example is amplification of the N-myc proto-oncogene, the copy number of N-myc is substantially elevated in some human tumors and far exceeds the normal two-copy state [1], [2]. In that case a proliferative phenotype will prevail by the sheer number of N-myc proteins produced as a result of the high copy number. Another possibility is miscoding of a fusion protein that render it unregulated and thus pro-cancerous. An event such as this is the hallmark of chronic myeloid leukemia - the BCR-ABL fusion (also termed the Philadelphia chromosome). The resulting fusion of ABL with BCR deregulates the normally well-controlled ABL protein, causing it to emit strong growth-promoting signals [3]. A third option is deregulation of a cancer-related gene by locating it near regulatory regions of another gene. Again, the myc proto-oncogene can serve as an example. Structural aberrations in the c-myc region on chr8q24 separate the c-myc gene from its normal promoter and place it under the control of one of three highly active promoters. Each of these promoters regulate an immunoglobin gene. Once expression is controlled by the antibody gene promoter, lymphoid cells in which these promoters are highly active will express large quantities of c-myc which will drive the relentless proliferation of cells, eventually leading to lymphomas [4].

In addition to well-defined molecular models reasoning the advantage potentially gained by certain aberrations, numerous regions in the genome are targets for aberrations that are associated with cancer but are yet to be rationalized molecularly. One such region is 17q. aberrations in 17q are linked to several cancer types such as lung [5], [6], [7], breast [8], prostate [9], [10], liver [11] and gastric cancer [10]. Structural alterations in 17q are most prominent in neuroblastoma cases and are linked to aggressive tumors [12], [13].

Using various methods, we have characterized a novel translocation occurring at the 17q24.3 locus within the BPTF gene in lung embryonic cells that strongly correlates with a pre-malignant phenotype. BPTF knock-down experiments restrict cell proliferation and thus suggest that aberrant expression of BPTF due to the translocation may contribute to malignancy. Lastly, we present clinical data in which the BPTF locus is amplified in human tumors and cancer cell lines proposing a role for aberrant BPTF expression in human cancer.

## Results

### Partial trisomy of 17q is correlated to a pre-malignant phenotype in lung embryonic cells

**Figure 1:**
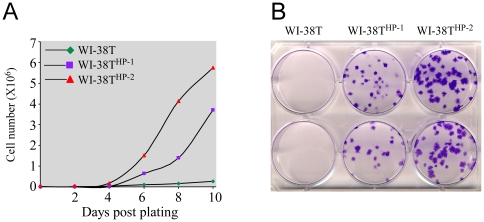
Highly proliferating WI-38T (WI-38T^HP-1/2^) exhibit pre-malignant phenotypes. (**A**) The growth curve of WI-38T and WI-38T^HP-1/2^ cells over 10 days is depicted. (**B**) The colony formation capability of WI-38T and WI-38T^HP-1/2^ is depicted.

Giving the strong correlation between chromosomal rearrangements and malignancy we examined the genomic integrity of cells derived from a normal, non-cancerous human tissue undergoing prolonged culturing. Three pools of WI-38 cells (lung embryonic fibroblasts cells) were separately immortalized by ectopic over-expression of the catalytic subunit of telomerase – hTERT, providing the cells with unlimited growth potential [14]. We were interested in monitoring the genomic structure of these cells with time without any further intervention that may facilitate any pro-cancerous advantage. Basically, only the first step in the long path leading to transformation was artificially introduced to the cells and then the cells were allowed to proliferate uninterrupted. The proliferation rate of the cells was fixed for 150 population doublings (PDLs) which stretched for roughly 250 days. At this stage a change in the rate of proliferation was observed. While one cell population (termed WI-38T) retained a fixed proliferation rate, the two other populations started to proliferate in an accelerated manner. These two cell populations were termed WI-38T^HP-1/2^ (for ‘Highly-Proliferating –1 and 2’) (Figure 1A). Furthermore, WI-38T^HP-1/2^ cells, in contrast to WI-38T, were able to form colonies *in vitro* (Figure 1B). Therefore, WI-38T^HP-1/2^ cells spontaneously obtained several pre-malignant traits – excessive proliferation, colony-formation ability and loss of contact inhibition. Of note, WI-38T^HP-2^ cells were more proficient in those traits than WI-38T^HP-1^ and thus constitute a more aggressive population.

**Figure 2:**
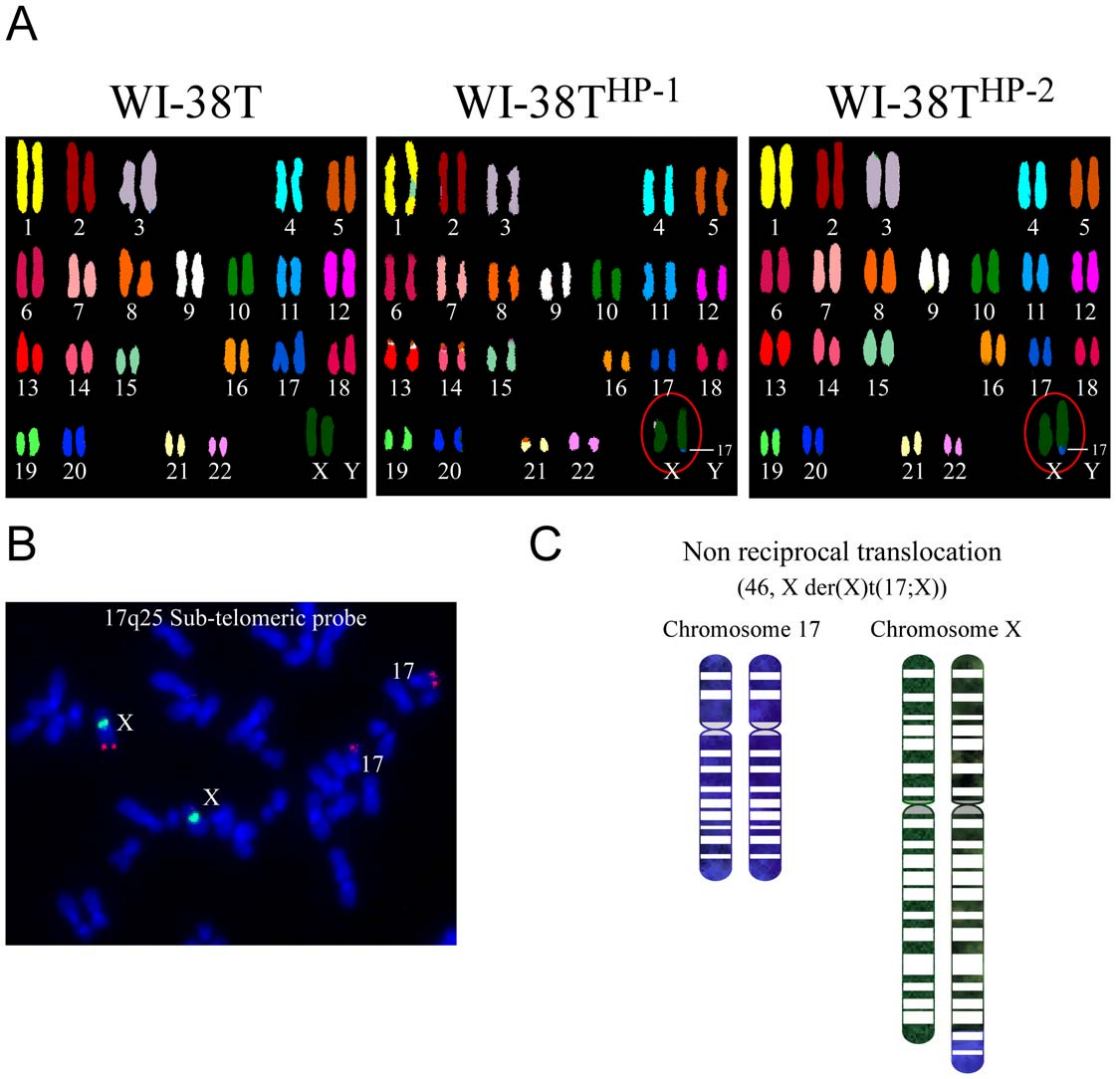
The WI-38T^HP-1/2^ cells harbor the der(X)t(X;17) translocation. (**A**) Spectral karyotyping (SKY) analysis of WI-38T and WI-38T^HP-1/2^ (passage 94) reveals a non-reciprocal translocation. The der(X)t(X;17) translocation is circled. (**B**) Fluorescence *in situ* hybridization (FISH) of 17q25 region in metaphase of WI-38T^HP-1^ cells. The probe detecting the sub-telomeric region of 17q25 (red) is visible on two copies of chr17 and on one copy of chrX. The chrX centromeric region is marked in green. (**C**) An illustration of the der(X)t(X;17) translocation.

Changes in chromosomal arrangement are considered as one of the triggers for pre-malignant phenotypes such as that presented by WI-38T^HP-1/2^. In order to examine the possibility that such rearrangements occurred in WI-38T^HP-1/2^ we performed a spectral karyotyping (SKY) analysis on parental WI-38, WI-38T and WI-38T^HP-1/2^. WI-38 and WI-38T cells exhibited a normal diploid karyotype (except for some abnormalities that were not consistent across the metaphases examined). In contrast, a non-reciprocal translocation der(X)t(X;17) was observed in 9 out of 10 of the examined metaphases of WI-38T^HP-1/2^ (Figure 2A and [14]). Fluorescence *in situ* hybridization (FISH) analysis of WI-38T^HP-1^ confirmed gain of 17q25 in the distal end of chromosome Xq (Figure 2B, on Figure 2C an illustration of the translocation is depicted). Taken together, these findings show a correlation between a pre-malignant phenotype of WI-38T^HP-1/2^ cells and the partial 17q trisomy that characterize these cells.

### The translocation breakpoint is mapped to the BPTF gene within the 17q24.3 region

**Figure 3:**
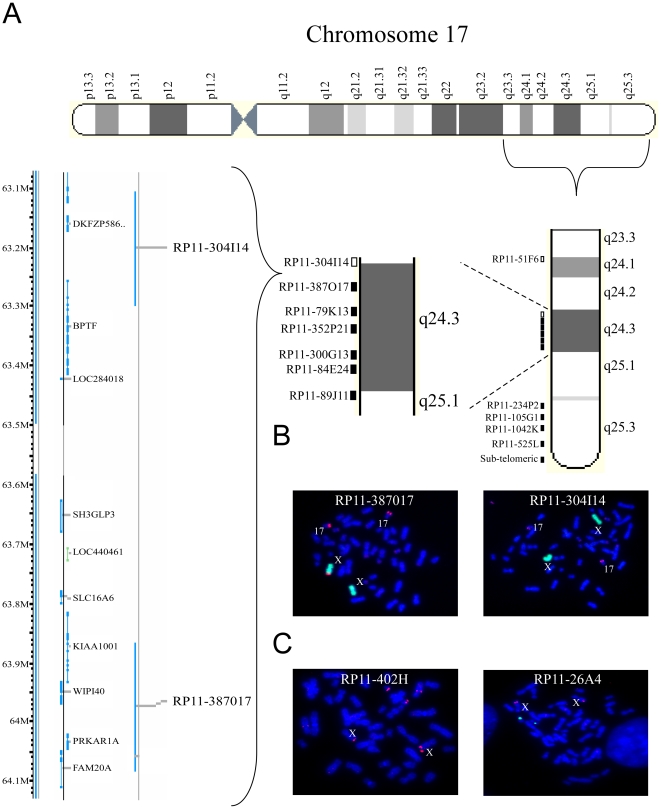
Mapping of the der(X)t(X;17) breakpoint. (**A**) A scheme of the bacterial artificial chromosome (BAC) probes used for the FISH analysis in order to localize the translocation. Filled squares represent probes detecting 17q material on both 17q and Xq, empty squares represent probes detecting 17q material only on 17q and not on Xq. A magnification of the breakpoint region is illustrated on the left-hand side of the panel. (**B**) The breakpoint region is localized in the 17q24.3 region. Fluorescence *in situ* hybridization analysis along the 17q arm of WI-38T^HP-1^ reveals the breakpoint region in the interval between two probes: RP11-387O17 (left-hand side – red) which is detected on chrX in addition to its normal position on chr17. In contrast, probe RP11-304I14 (right-hand side – red) is detected only on the two normal copies of chr17 and not on chrX. ChrX material is marked in green. (**C**) No aberrations in chromosome Xq material are detected. Fluorescence *in situ* hybridization along Xq arm. The two probes detecting the most distant area of the Xq arm (RP11-304H and RP11-26A - red) are visible only on the two copies of chrX and not on chr17. The chr17 centromeric region is marked in green.

In order to more specifically localize the chromosomal breakpoint we performed FISH analysis using consecutive tandem bacterial artificial chromosome (BAC) probes, starting with a probe detecting the sub-telomeric region of 17q, followed by a probe detecting a region less distant from the centromere and so on (Figure 3A). A total of 11 probes (starting with the sub-telomeric probe to probe RP11-387O17) were detected both on the full chromosome 17 and on Xqter of WI-38T^HP-1^ cells, suggesting a partial trisomy of these genomic areas, evidenced by probes on chromosome X. In contrast, the probes designed to identify regions 17q24.3 and 17q24.1 were only detected on the full chromosome 17 (probes RP11-304I14 and RP11-51F6 respectively). This probe-signal pattern mapped the breakpoint to the 17q24.3 region enclosed between probes RP11-387O17 and RP11-304I14 (Figure 3A, B). The gap between these two probes stretches for an area of about 0.5Mbp. Six coding sequences reside within this area: BPTF, LOC284018, SH3GLP3, LOC440461, SLC16A6 and KIAA1001. No rearrangements of Xq fragments were detected as the two most distal probes detecting Xqter, RP11-402H and RP11-26A4, revealed normal ploidy (Figure 3C).

**Figure 4:**
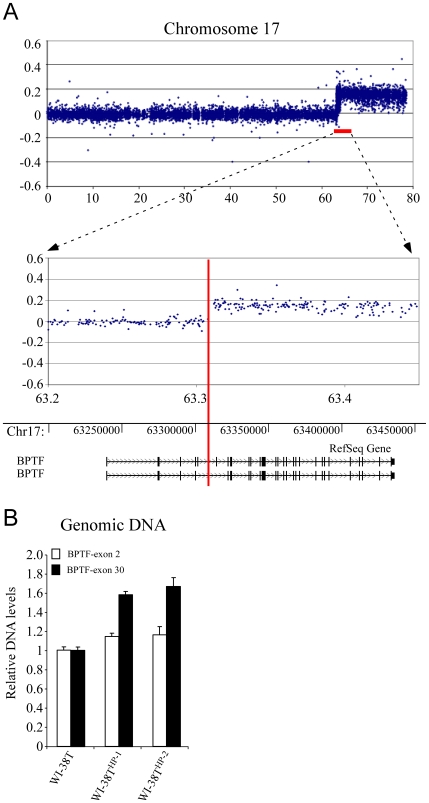
Localization of the breakpoint region within the fourth intron of BPTF gene. (**A**) DNA samples from primary WI-38 and WI-38T^HP-1^ were labeled and hybridized to Agilent custom arrays covering the 0.5-Mb region (the distance between probe RP11-387O17 and RP11-304I14) on chromosome 17 with an average probe spacing of 0.5 kb. (**B**) Quantitative Real-Time PCR analysis on genomic DNA from WI-38T^HP-1/2^ in the BPTF gene revealed a 1.5 fold increase in BPTF genomic dosage compared to WI-38T cells. Two sets of primers were used to detect the gene dosage of BPTF. One set annealing to the 5-prime end of the BPTF gene (BPTF-exon 2) and the second set annealing to the 3-prime end of it (BPTF-exon 30).

**Figure 5:**
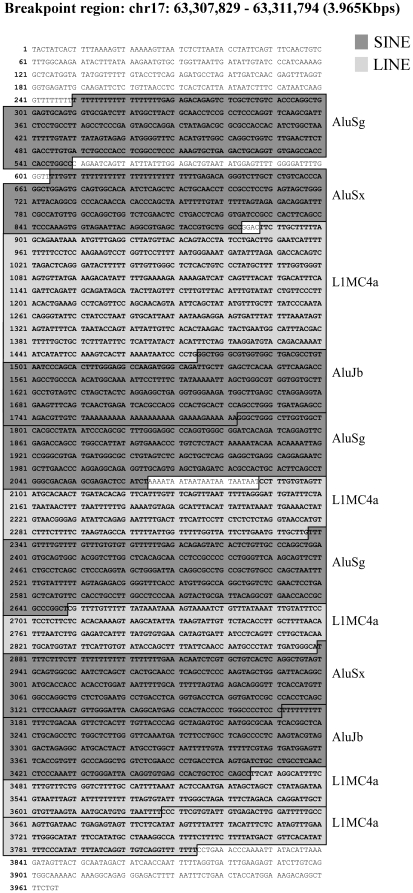
The breakpoint region is highly enriched with repetitive elements. Twelve repetitive elements in the ∼4 Kb breakpoint region are depicted. Short interspersed nuclear elements (SINEs) are marked in dark grey and long interspersed nuclear elements (LINEs) are marked in light grey (adopted from the UCSC genome browser).

After confining the area of the breakpoint to a specific chromosomal region we set out to characterize the exact location of this breakpoint. To this end, we performed a comparative genomic hybridization assay (CGH). We have designed a custom CGH array in which focus was put on the breakpoint region. This 0.5Mbp-long region in 17q was subjected to a large number of probes giving an average distance of 500 bp between two probes. Chromosome Xq was also scanned with a high-resolution probe design. As shown in Figure 4A the CGH output showed a clear-cut 1.5 fold increase in probe intensity in the 17q region starting from the fourth intron of the BPTF gene, stretching on to the sub-telomeric end of 17q. This 1.5 fold increase in the signal suggests a gain of the detected chromosomal region (i.e. partial trisomy of 17q). In agreement with the FISH analysis, the CGH output represented normal ploidy of Xq (data not shown). Therefore, we have succeeded in mapping the breakpoint region to a 4 Kb-long region within the fourth intron of BPTF (chr17: 63,307,829 - 63,311,794). This region is densely packed with 12 repetitive elements (7 SINEs and 5 LINEs – Figure 5). As repetitive elements are known to be fragile and therefore considered hot spots for breakpoints it is conceivable that these elements contributed to this rearrangement event. Further indication of the breakpoint location was achieved by quantitative real time PCR (QRT-PCR) performed with primers annealing to genomic regions of the BPTF gene. The level of a PCR product of an amplified 5′ genomic region of BPTF was comparable in WI-38T and WI-38T^HP-1/2^. In contrast, when amplifying a 3′ genomic region of BPTF a ∼1.5 fold increase in the level of the PCR product was observed in WI-38T^HP-1/2^ relative to its level in WI-38T (Figure 4B).

### Knock-down of excessive BPTF negates the pre-malignant phenotype of WI-38T^HP-1/2^

**Figure 6:**
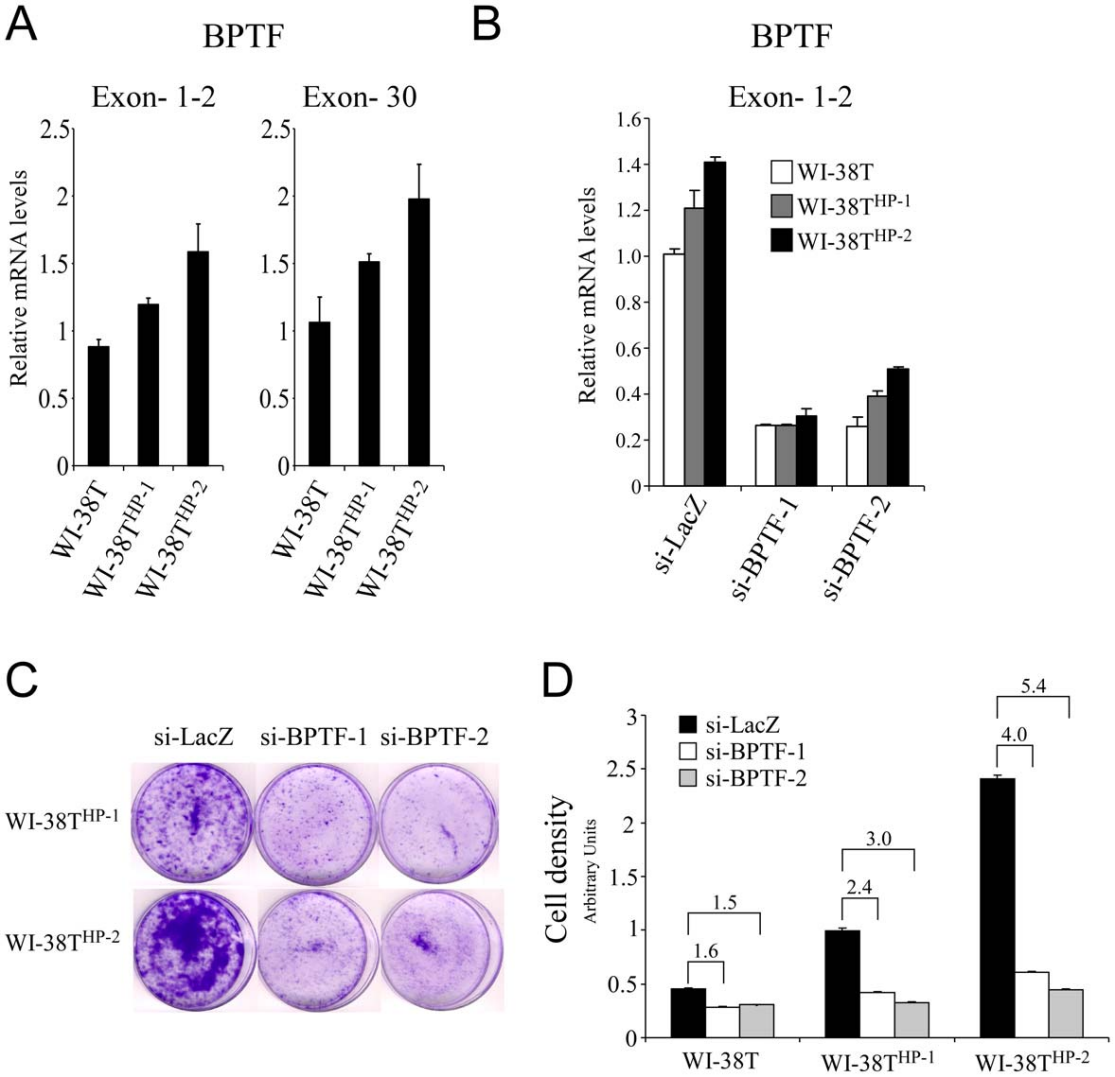
BPTF knock-down restricts proliferation. (**A**) Quantitative Real-Time PCR analysis on mRNA from WI-38T and WI-38T^HP-1/2^ in the BPTF gene revealed an increase in the endogenous BPTF mRNA in WI-38T^HP-1/2^ cells. (**B**) Quantitative Real-Time PCR on mRNA from WI-38T and WI-38T^HP-1/2^ following BPTF knock-down. (si-BPTF-1 targeted the translocated region while si-BPTF-2 targeted the endogenous region of BPTF). (**C**) Growth inhibition of cells by knocking-down BPTF is more efficient in WI-38T^HP-2^ cells. Depicted is crystal violet staining of cells five days following si-RNA transfection. (**D**) Quantification of the crystal violet from panel C, numbers above bars describe the fold change, depicted is a representative from three independent experiments.

Next, we were interested in assessing BPTF mRNA level. We used two primer sets in a QRT-PCR analysis, one set detected the first two exons of BPTF, an area that was not duplicated, and the second set detected the last exon of BPTF, an area duplicated as a result of the translocation. The results were intriguing as both the non-duplicated region and the duplicated region were elevated (Figure 6). The fact that the non-duplicated region is also up-regulated transcriptionaly suggests that the translocation affects the regulation of the endogenous BPTF transcript and not only the rearranged area. In addition, the mRNA level of BPTF in WI-38T^HP-2^ was highest whereas WI-38T^HP-1^ exhibited an intermediate level of BPTF mRNA. This finding complements the data shown in Figure1A and B where WI-38T^HP-2^ presented the most aggressive phenotype and a milder phenotype was presented in WI-38T^HP-1^. Therefore, a correlation exists between BPTF mRNA level and an aggressive phenotype.

In order to elucidate the effect of the BPTF translocation WI-38T and WI-38T^HP-1/2^ cells were transfected with small-interfering oligonucleotides targeting BPTF (si-BPTF) (si-BPTF-1 targeted the translocated region while si-BPTF-2 targeted the endogenous region of BPTF) causing a reduction in BPTF mRNA level (Figure 6B). Interestingly, when the translocated region of BPTF was knocked-down (si-BPTF-1), BPTF level was equally reduced in all three populations, however, when the non-translocated region of BPTF was knocked-down (si-BPTF-2) BPTF was reduced, but the expression pattern of BPTF resembled that of the control (in which WI-38T^HP-2^ exhibit the highest levels of BPTF followed by WI-38T^HP-1^ and then WI-38T). When the growth capacity of WI-38T and WI-38T^HP-1/2^ was assessed using crystal violet staining an attenuated growth rate was observed in cells treated with either of the two types of si-BPTF (Figure 6C and 6D). A similar pattern of growth attenuation was observed using a cell counter (data not shown). Importantly, a strong correlation between BPTF levels and the growth inhibition effect was noticed as the most pronounce effect in growth inhibition was witnessed in WI-38^HP-2^ while an intermediate effect in WI-38T^HP-1^ and a mild effect in WI-38T were witnessed, indicating that BPTF is one of the major players that contribute to the pre-malignant phenotypes observed. Taken together, these data support a notion that translocation-dependent dysregulation of BPTF is responsible for the pre-malignant phenotype of WI-38T^HP-1/2^.

**Figure 7:**
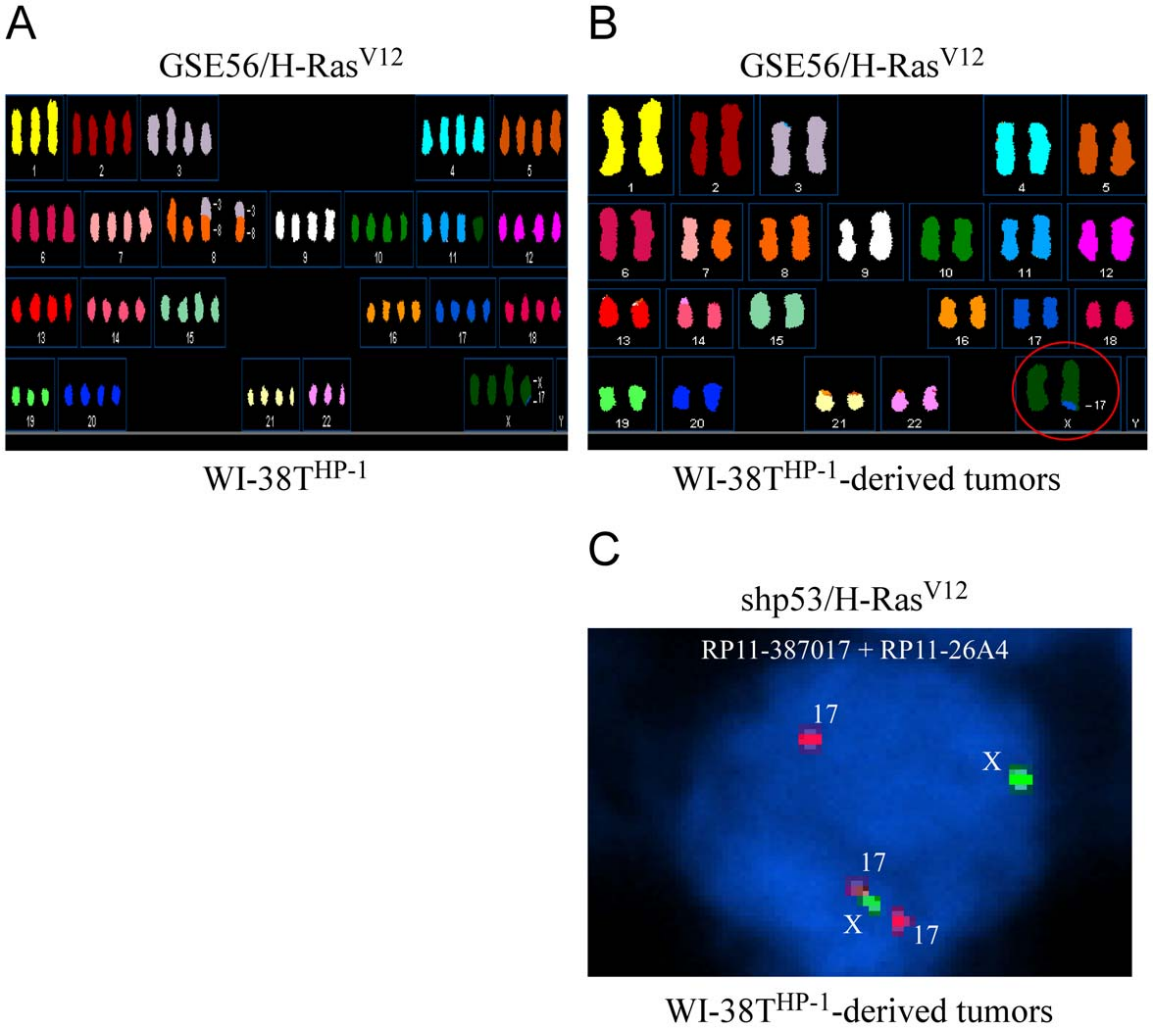
The der(X)t(X;17) translocation is selected in tumors-derived from WI-38T^HP-1^ cells harboring the GSE56 and H-Ras^V12^ constructs. (**A**) The WI-38T^HP-1^ cells that express the GSE56 and oncogenic H-Ras^V12^ exhibit genomic instability. Spectral karyotyping (SKY) analysis of WI-38T^HP-1^ cells which were introduced with GSE56 and oncogenic H-Ras^V12^ reveals genomic instability manifested by polysomy and several genomic aberrations. (**B**) A SKY analysis of WI-38T^HP-1^ tumor-derived cells is shown, the der(X)t(X;17) translocation is circled. (**C**) A FISH analysis on WI-38T^HP-1^ tumor-derived cells that harbor a shRNA against p53 (shp53) and oncogenic H-Ras^V12^. The probe detecting chr17q24.3 (RP11-387O17 - red) is visible on two copies of chr17 and on one copy of chrX. A probe detecting the most telomeric X chromosomal region (RP11-26A4) is marked in green.

The significance of the translocation was further highlighted when WI-38T^HP-1^ cells were introduced with GSE56 (a dominant negative peptide targeting the p53 tumor suppressor) and a constitutively active Ras oncoprotein (H-Ras^V12^) which resulted in many chromosomal aberrations as visualized by SKY (Figure 7A). This aggressive sub-population of WI-38T^HP-1^ resulted in full-grown tumors when 10^7^ cells were subcutaneously injected to five nude mice [15]. The only translocation present in all five tumors examined as visualized by SKY was the original der(X)t(X;17) translocation (Figure 7B) while none of the other rearrangements present in the cells prior to injection were detected. The fact that this translocation, after constant selection pressure subjected *in vivo*, was the only chromosomal aberrancy inhabiting the tumor cells alludes to its advantageous tumorigenic properties to the cells. Furthermore, the same experimental setup was repeated but with a different p53-inactivating method. In Figure 7C a FISH analysis of a mouse-originated tumor is shown in which p53 was inactivated by short-hairpin RNA targeting p53 (sh-p53). Here, the probe detecting the translocation (RP11-387O17) showed that the rearrangement event existed in the tumor.

### Gain of the BPTF locus is a frequent aberrancy in neuroblastomas, lung adenocarcinomas and in various cancer cell lines

**Figure 8:**
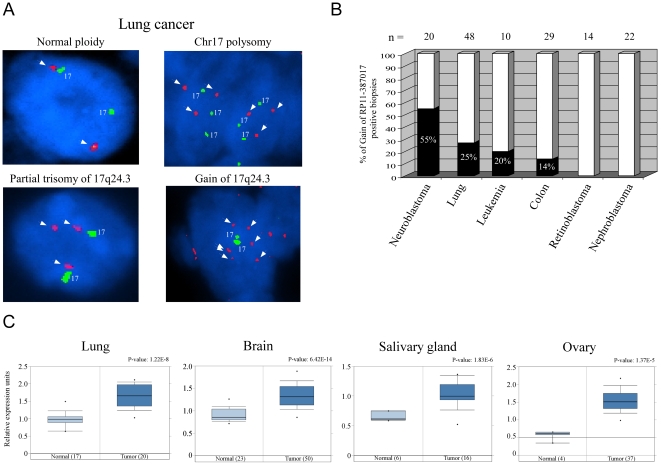
Gain of the BPTF genomic locus and mRNA levels in human tumors. (**A**) Representative pictures of the various chromosomal rearrangements in the BPTF locus in six human tumor types. FISH analysis using probe RP11-387O17 (detecting 17q24.3 region in the BPTF locus) and a probe detecting chr17 centromeric material to distinguish between polysomy and gain of the BPTF locus. The nuclei presented are of lung adenocarcinoma cells and are representative of the different probe patterns detected in the other tumors (colon, neuroblastomas and leukimias). a) normal ploidy. b) chr17 polysomy. c) partial trisomy of 17q24.3. d) gain of 17q24.3. (**B**) A graph depicts the percentage of samples containing gain of 17q24.3 (a sample in which over 30% of cells exhibit gain was considered a positive sample) as assessed by FISH of a paraffin tissue array containing 143 tumor samples. (**C**) Expression of BPTF in several human tumors. Depicted is the relative expression of BPTF in several human cancers compared to normal tissues as obtained from the ONCOMINE database.

In order to evaluate the gene dosage of BPTF in human tumors, commercially available tumor arrays containing 143 tumor samples from six human cancer types (neuroblastomas, lung adenocarcinomas, nephroblastomas, leukemias, colon cancer and retinoblastomas) were assessed. FISH analysis using probe RP11-387O17 (detecting 17q24.3 region in the BPTF locus) and a probe detecting chr17 centromeric material to distinguish between polysomy and gain of the BPTF locus was performed. Several genomic aberrations were observed in the BPTF locus in the various tumor types such as polysomy, partial trisomy of 17q24.3 and gain of 17q24.3 (Figure 8A). This analysis revealed that 55% of neuroblastomas and 27% of lung tumors exhibited gain of 17q24.3, followed by leukemia and colon cancer in which 20% and 14% of the tumors examined presented gain of 17q24.3, respectively. No such abnormalities were detected in retinoblastomas and nephroblastomas (Figure 8B). Importantly, in 67% of the BPTF-positive lung tumors cases, gain of the 17q24.3 locus was associated with poor prognosis (grade III), while only 37% of the cases were graded either II or I. Regarding the other tumor types, grading was either not available (neuroblastomas, nephroblastomas, leukemias, retinoblastomas) or was not significant due to small sample size. In accordance with that, an *in silico* analysis using the ONCOMINE database [16] revealed significant elevation in BPTF mRNA levels in several human cancers (lung, brain, ovary and salivary gland) (Figure 8C).

To further investigate the copy number status of the BPTF locus in different cancer types, we analyzed the BPTF locus status in the 60 cell lines composing the NCI-60 panel [17]. We inspected several genomic locations inside the BPTF locus using the NCI-60 CGH dataset described in [18]. For all these genomic locations we observed that 25 of the 60 cell lines contained an amplified BPTF locus (42%) while only 2 had deletions in this locus. We also note that amplifications of the 17qter are in general prevalent in the NCI-60 cell lines.

The fact that gain of the BPTF locus and up-regulation of the BPTF gene epitomize many human cancers indicates that our cellular model with a defined 17q24.3 translocation located within BPTF causing an elevation in endogenous BPTF could shed light on cancer-associated 17q translocations and that BPTF could be an important player in development of those malignancies.

## Discussion

In this work we have shown that the 17q24.3 region is translocated in human lung embryonic fibroblasts and that this event perfectly correlated to a high proliferation rate. The translocation breakpoint was mapped to the BPTF gene suggesting a role for BPTF in proliferation. These data was strengthened by BPTF knock-down experiments showing inhibition of proliferation. Unfortunately, attempts made at cloning and over-expressing the BPTF gene were unfruitful due to its gigantic mRNA (∼11 Kb). In addition, it was impossible to asses the translocated BPTF protein levels since the available antibodies against BPTF did not produce a band in several western blot analyses that we performed (Orbigen, cat# PAB-02251 and Bethyl cat# A300-973A). Nonetheless, when the BPTF locus was monitored in human cancers, a clear gain of its locus was noticed.

As mentioned above, many types of human cancer demonstrate rearrangements of the 17q region. Partial gain of chromosome arm 17q is the most abundant genetic alteration in neuroblastoma and constitutes the strongest genetic factor for adverse prognosis [12]. It is assumed that one or more genes on 17q contribute to neuroblastoma by a gene dosage effect. Furthermore, studies aimed at mapping 17q breakpoints in neuroblastoma and assessing their significance concluded that genes mapping between 17q21 and 17qter are important for neuroblastoma pathogenesis [13], [19], [20], [21]. Given that neuroblastoma is an embryonic tumor it is tempting to speculate that the translocation revealed in WI-38 cells, which are derived from an embryonic origin, is analogous to translocations occurring in neuroblastoma cases.

Attempts made at identifying the gene or genes in the 17q region responsible for neuroblastoma promotion were unfruitful. The human Bromodomain PHD finger Transcription Factor gene (BPTF) shown here to be translocated resides within this breakable region. This gene is transcribed into two different messengers and proteins: the 2,781 amino acid long BPTF protein [22] and the shorter fetal ALZ50-reactive clone 1 (FAC1) which consists of only 801 N-terminal amino acids of BPTF and lacks the bromodomain [23]. Several elements in the BPTF sequence imply to transcriptional regulatory abilities such as a DDT DNA binding domain [24], a PHD zinc finger, a glutamine-rich acidic domain and a bromodomain [22]. Indeed, FAC1 was suggested to regulate transcription by directly binding DNA [25] and BPTF was shown to be involved in chromatin remodeling [26], [27].

Cancer is a disease characterized by deregulation of transcriptional programs and the majority of genes considered as oncogenes or tumor suppressors are involved in the transcriptional aspect of tumorigenesis. As transcription-regulators, several bromodomain-containing proteins have been associated with malignancy and translocation breakpoints have been detected in some of the genes encoding these proteins: HRX/ALL-1 [28], [29], TIF1 [30] and RFG7 [31]. These findings, combined with our observations that the chromosomal region harboring the BPTF gene is translocated and that this translocation strongly correlates with a pre-malignant phenotype implies for the involvement of BPTF/FAC1 in regulating cellular proliferation.

The means by which the translocated BPTF affects proliferation are yet to be elucidated, in line with reported cases of deregulated genes and proteins due to chromosomal aberrations, several scenarios can be proposed. The translocation event may cause BPTF to be translated as a truncated protein. Alternatively, the protein function of BPTF/FAC1 may remain unchanged but its transcriptional regulation is modified due to its new location in the genome. A scenario of a fusion protein seems less plausible since no aberrations were detected on chrX. However, deregulation of endogenous BPTF due to a truncated protein is conceivable since an up-regulation of endogenous BPTF was observed in cells harboring the translocation. Such regulation could be mediated for example by squelching of a BPTF transcriptional repressor (as in dominant negative mechanisms) by the miscoded BPTF.

A case where a deregulated BPTF/FAC1 can affect proliferation can be conceived: the physical interaction between FAC1 and the Myc-associated zinc finger protein (ZF87/MAZ) reduces ZF87/MAZ activity [32]. Interestingly, ZF87/MAZ is a negative regulator of the c-myc proto-oncogene [33]. Therefore, an indirect positive regulation of c-myc by FAC1 is feasible where FAC1 inhibits repressional regulation of the c-myc promoter by ZF87/MAZ. This possible regulation of BPTF/FAC1 over c-myc comes in accordance with the observation that WI-38T^HP1^ exhibit high c-myc levels [14] and the finding presented in Figures 1 and 6 where BPTF promotes a proliferative phenotype as knock-down of BPTF restricts proliferation.

A situation in which a pro-growth gene (as BPTF seems to be) is aberrantly expressed due to structural rearrangements of the genome grants the cell harboring this aberrancy with a growth advantage that allows it to overcome the natural obstacles preventing transformation. After showing the connection between the aforementioned translocation and a growth advantage provided to the cells, and mapping the translocation breakpoint to BPTF, we suggest that BPTF could play a cancer-promoting role in neoplasms harboring chromosomal aberrations in 17q.

## Materials and Methods

### Cell culture and transfections

The immortalized primary human embryonic lung fibroblasts (WI-38T and WI-38T^HP-1/2^) were established and maintained as described [14]. Knock-down of BPTF was conducted by transfection with specific oligo-nucleotides using DharmaFECT3 reagent (both from DHARMACON, Lafayette, CO). In the experiments described in Figures 6B and 6C cells were plated in 6 cm plates −4×10^4^ in 6B or 1×10^4^ in 6C and were transfected 48 h later with small interfering RNA targeting BPTF or LacZ as a control. In Figure 6C cells were stained with crystal violet five days post transfection.

### Quantification of cell growth and colony formation assay

For the quantification of cells depicted in Figure 1A cells were plated in 24-well plates (2×10^5^ cells per well) and were counted every 24 h using Countess^TM^ automated cell counter (Invitrogen). For the colony formation assay depicted in Figure 1B cells were plated in 6-well plates (1×10^2^ cells per plate) and stained with crystal violet 14 days later.

### DNA and RNA isolation and Quantitative Real Time-PCR (QRT-PCR)

For DNA extraction, cell pellet was resuspended in 400 µl Buffer A (100 mM Tris-HCl, pH 7.5, 100 mM EDTA, 100 mM NaCl, 0.5% SDS) and incubated at 65° for 30 minutes. 800 µl LiCl/KAc solution was added followed by incubation on ice for 10 minutes. Next, lysate was centrifuged for 15 minutes at RT. 1 ml of supernatant was transferred and 600 µl isopropanol was added, followed by 15 minutes centrifugation at RT. Pellet was washed with 70% ethanol and resuspended in 50 µl TE.

Total RNA was isolated using NucleoSpin kit (Macherey-Nagel) according to the manufacturer's protocol. A 2 µg aliquot of the total RNA was reverse transcribed using Bio-RT (9597, BIO LAB) and random hexamer primers. QRT-PCR was done with ABI 7300 instrument (Applied Biosystems, Foster City, CA) using Platinum SYBR Green qPCR SuperMix (Invitrogen,Carlsbad, CA). Two sets of primers were used to measure the genomic dosage of BPTF. The first set annealing to the 5-prime end of the BPTF gene (BPTF-exon 2- sense-GTG CTG CGG GTG TAC TGT GA, anti-sense- AGG AAG AAC GTG ATG GTA CTC CTT). The second set annealing to the 3-prime end of BPTF (BPTF-exon 30- sense-CAC TAA GCT GTC AGC TCT GCT CTT, anti-sense- TCG GCT CTG AGC TGC TCTT T). Two sets of primers were used to measure the mRNA dosage of BPTF. The first set annealing to the 5-prime end of the BPTF gene (BPTF-exon 1-2- sense- CAG CAG CAC TCC AGG TAG GC anti-sense-GGA GAA CGA GGC CGA TGT ACT). The second set annealing to the 3-prime end of BPTF (BPTF-exon 30- sense-CAC TAA GCT GTC AGC TCT GCT CTT, anti-sense- TCG GCT C TGA GCT GCT CTT T). cDNA levels were normalized to *GAPDH* (sense-ACC CAC TCC TCC ACC TTT GA, anti-sense- CTG TTG CTG TAG CCA AAT TCG T).

### Tissue arrays and anaphase fluorescence in situ hybridization (FISH) analyses

The tissue arrays were purchased from Biomax (www.biomax.us), cat# - MC803 and MC2081. These arrays are IRB and HIPPA approved. The paraffin sections contained 143 tumor samples from six human cancer types (neuroblastomas, lung adenocarcinomas, nephroblastomas, leukemias, colon cancer and retinoblastomas). FISH was performed using combination of probes RP11-387O17 detecting 17q24.3 region in the BPTF locus labeled with green fluochrome and a probe detecting chr17 centromeric probes -labeled with red flurochrome (for labeling see metaphase FISH). The slides were first deparaffinized with xylene and then incubated in 0.1 mM citric acid pH 6 twice for 5 min in a microwave. Following digestion with 4 mg/ml pepsin (Sigma P-7012) for 15 min at 37C°, the slides were rinsed in 2XSSC for 5 min at room temperature and air dried. 10 µl probes were added to the slides and covered with a coverslip and sealed with rubber cement. Slides were co-denatured in an Omnislide *in situ* hybridization system (Hybaid limited) at 80C° for 5 min and then hybridized at 37C° for 16 hrs. The slides were washed according to probe specification and counter stained with DAPI (4′–6′-diamidine-2-phenylindole). The samples were viewed with a Provis-Olympus fluorescent microscope equipped with a 100W mercury lamp and DAPI, orange, green, dual orange/green filters. Images were captured using the QUIPS XL Genetics Workstation (Vysis). FISH samples were evaluated by two microscopists. At least 100 nuclei were examined for each case.

### Metaphase fluorescence in situ hybridization (FISH) analyses

Metaphase spread was prepared from the cell lines using standard techniques. The probes were labeled by nick translation using a Nick Translation Reagent Kit with Spectrum Orange or Spectrum Green according to the instruction provided by the supplier (Abbot Molecular Inc.). The probes were ethanol precipitated and resuspended each at 10 µl of hybridization solution (50% Formamide, 2XSSC and 10% Dextran Sulfate). Slides were denatured in 70% Foromamide /2XSSC at 72C°, the probes were denatured separately at 75C° for 7 minutes and applied to the slides for hybridization at 37C° over night. After washing at 72C° in 0.4XSSC for 2 minutes, the slides were stained with 4,6-diamidino-2 phenylindole (DAPI) in antifade medium.

### Spectral karyotyping (SKY) analysis

Chromosome specific libraries generated by PCR from flow-sorted human chromosomes were labeled with one of five different nucleotides conjugated to five different dyes (FITC, Rodamine, Texas Red, Cy5 and Cy5.5) or a combination thereof. All 24 chromosomes libraries were hybridized simultaneously to the metaphases. After washing the slides were stained with 4,6-diamidino-2 phenylindole (DAPI) in antifade medium. From each case between 5-10 metaphases were captured using a fluorescent microscope (Olympus BX51) using a custom designed triple band pass filter (SKY, Chroma Technology, Battleboro, VT) that allows simultaneous excitation of all dyes and measurement of their emission spectra. The discrimination between the different spectra was done using the SD300 spectral bio-imaging system. The system enables the measurement of the full visible light spectrum at each pixel of the image by using a Sagnac interferometer. A classification algorithm was used to differentiate between different spectra in the image and to assign pseudocolors to all the pixels which have similar spectral characteristics. The DAPI image was captured separately and inverted to give a G-banding like pattern. The chromosomes were then sorted automatically into a karotype table.

### Comparative genomic hybridization *(*CGH*)*

DNA samples from primary WI-38 and WI-38T^HP-1^ were labeled and hybridized to Agilent custom CGH arrays covering the 0.5-Mb region (the distance between probe RP11-387O17 and RP11-304I14) on chromosome 17 with an average probe spacing of 0.5 kb (www.agilent.com) using Agilent standard CGH protocols [34]. A custom made oligonucleotide array designed for CGH contained 12099 chrX and 9225 chr17 60-mer probes. These probes included both coding and noncoding sequences on these chromosomes. Probes design, for 60-mer probes, was performed using the methods described in [35]. Design takes into account uniqueness in the genome, thermodynamic uniformity and seeks to maximize uniformity of coverage under these constraints. The array design is uploaded for manufacturing using Agilent's eArray software.

## References

[1] Schwab M, Alitalo K, Klempnauer KH, Varmus HE, Bishop JM (1983). Amplified DNA with limited homology to myc cellular oncogene is shared by human neuroblastoma cell lines and a neuroblastoma tumour.. Nature.

[2] Seeger RC, Brodeur GM, Sather H, Dalton A, Siegel SE (1985). Association of multiple copies of the N-myc oncogene with rapid progression of neuroblastomas.. N Engl J Med.

[3] Faderl S, Talpaz M, Estrov Z, O'Brien S, Kurzrock R (1999). The biology of chronic myeloid leukemia.. N Engl J Med.

[4] Leder P, Battey J, Lenoir G, Moulding C, Murphy W (1983). Translocations among antibody genes in human cancer.. Science.

[5] Choi JS, Zheng LT, Ha E, Lim YJ, Kim YH (2006). Comparative genomic hybridization array analysis and real-time PCR reveals genomic copy number alteration for lung adenocarcinomas.. Lung.

[6] Wong MP, Fung LF, Wang E, Chow WS, Chiu SW (2003). Chromosomal aberrations of primary lung adenocarcinomas in nonsmokers.. Cancer.

[7] Yen CC, Liang SC, Jong YJ, Chen YJ, Lin CH (2007). Chromosomal aberrations of malignant pleural effusions of lung adenocarcinoma: different cytogenetic changes are correlated with genders and smoking habits.. Lung Cancer.

[8] Beser AR, Tuzlali S, Guzey D, Dolek Guler S, Hacihanefioglu S (2007). HER-2, TOP2A and chromosome 17 alterations in breast cancer.. Pathol Oncol Res.

[9] Levin AM, Machiela MJ, Zuhlke KA, Ray AM, Cooney KA (2008). Chromosome 17q12 variants contribute to risk of early-onset prostate cancer.. Cancer Res.

[10] Sun J, Purcell L, Gao Z, Isaacs SD, Wiley KE (2008). Association between sequence variants at 17q12 and 17q24.3 and prostate cancer risk in European and African Americans.. Prostate.

[11] Raidl M, Pirker C, Schulte-Hermann R, Aubele M, Kandioler-Eckersberger D (2004). Multiple chromosomal abnormalities in human liver (pre)neoplasia.. J Hepatol.

[12] Bown N, Cotterill S, Lastowska M, O'Neill S, Pearson AD (1999). Gain of chromosome arm 17q and adverse outcome in patients with neuroblastoma.. N Engl J Med.

[13] Lastowska M, Cotterill S, Bown N, Cullinane C, Variend S (2002). Breakpoint position on 17q identifies the most aggressive neuroblastoma tumors.. Genes Chromosomes Cancer.

[14] Milyavsky M, Shats I, Erez N, Tang X, Senderovich S (2003). Prolonged culture of telomerase-immortalized human fibroblasts leads to a premalignant phenotype.. Cancer Res.

[15] Milyavsky M, Tabach Y, Shats I, Erez N, Cohen Y (2005). Transcriptional programs following genetic alterations in p53, INK4A, and H-Ras genes along defined stages of malignant transformation.. Cancer Res.

[16] Rhodes DR, Yu J, Shanker K, Deshpande N, Varambally R (2004). ONCOMINE: a cancer microarray database and integrated data-mining platform.. Neoplasia.

[17] Weinstein JN, Myers TG, O'Connor PM, Friend SH, Fornace AJ (1997). An information-intensive approach to the molecular pharmacology of cancer.. Science.

[18] Lipson D, Aumann Y, Ben-Dor A, Linial N, Yakhini Z (2006). Efficient calculation of interval scores for DNA copy number data analysis.. J Comput Biol.

[19] Bown N, Lastowska M, Cotterill S, O'Neill S, Ellershaw C (2001). 17q gain in neuroblastoma predicts adverse clinical outcome. U.K. Cancer Cytogenetics Group and the U.K. Children's Cancer Study Group.. Med Pediatr Oncol.

[20] Meddeb M, Danglot G, Chudoba I, Venuat AM, Benard J (1996). Additional copies of a 25 Mb chromosomal region originating from 17q23.1-17qter are present in 90% of high-grade neuroblastomas.. Genes Chromosomes Cancer.

[21] Van Roy N, Laureys G, Van Gele M, Opdenakker G, Miura R (1997). Analysis of 1;17 translocation breakpoints in neuroblastoma: implications for mapping of neuroblastoma genes.. Eur J Cancer.

[22] Jones MH, Hamana N, Shimane M (2000). Identification and characterization of BPTF, a novel bromodomain transcription factor.. Genomics.

[23] Zhu P, Bowser R (1996). Identification and analysis of the complete cDNA sequence for the human FAC1 gene.. Biochim Biophys Acta.

[24] Doerks T, Copley R, Bork P (2001). DDT -- a novel domain in different transcription and chromosome remodeling factors.. Trends Biochem Sci.

[25] Jordan-Sciutto KL, Dragich JM, Rhodes JL, Bowser R (1999). Fetal Alz-50 clone 1, a novel zinc finger protein, binds a specific DNA sequence and acts as a transcriptional regulator.. J Biol Chem.

[26] Li H, Ilin S, Wang W, Duncan EM, Wysocka J (2006). Molecular basis for site-specific read-out of histone H3K4me3 by the BPTF PHD finger of NURF.. Nature.

[27] Wysocka J, Swigut T, Xiao H, Milne TA, Kwon SY (2006). A PHD finger of NURF couples histone H3 lysine 4 trimethylation with chromatin remodelling.. Nature.

[28] Gu Y, Nakamura T, Alder H, Prasad R, Canaani O (1992). The t(4;11) chromosome translocation of human acute leukemias fuses the ALL-1 gene, related to Drosophila trithorax, to the AF-4 gene.. Cell.

[29] Tkachuk DC, Kohler S, Cleary ML (1992). Involvement of a homolog of Drosophila trithorax by 11q23 chromosomal translocations in acute leukemias.. Cell.

[30] Le Douarin B, Zechel C, Garnier JM, Lutz Y, Tora L (1995). The N-terminal part of TIF1, a putative mediator of the ligand-dependent activation function (AF-2) of nuclear receptors, is fused to B-raf in the oncogenic protein T18.. Embo J.

[31] Klugbauer S, Rabes HM (1999). The transcription coactivator HTIF1 and a related protein are fused to the RET receptor tyrosine kinase in childhood papillary thyroid carcinomas.. Oncogene.

[32] Jordan-Sciutto KL, Dragich JM, Caltagarone J, Hall DJ, Bowser R (2000). Fetal Alz-50 clone 1 (FAC1) protein interacts with the Myc-associated zinc finger protein (ZF87/MAZ) and alters its transcriptional activity.. Biochemistry.

[33] Izzo MW, Strachan GD, Stubbs MC, Hall DJ (1999). Transcriptional repression from the c-myc P2 promoter by the zinc finger protein ZF87/MAZ.. J Biol Chem.

[34] Barrett MT, Scheffer A, Ben-Dor A, Sampas N, Lipson D (2004). Comparative genomic hybridization using oligonucleotide microarrays and total genomic DNA.. Proc Natl Acad Sci U S A.

[35] Lipson D, Yakhini Z, Aumann Y (2007). Optimization of probe coverage for high-resolution oligonucleotide aCGH.. Bioinformatics.

